# A Modified Residual-Based RAIM Algorithm for Multiple Outliers Based on a Robust MM Estimation

**DOI:** 10.3390/s20185407

**Published:** 2020-09-21

**Authors:** Wenbo Wang, Ying Xu

**Affiliations:** 1 Aerospace Information Research Institute, Chinese Academy of Science, Beijing 100864, China; xuying@aircas.ac.cn; 2 School of Electronic, Electrical and Communication Engineering, University of Chinese Academy of Sciences, Beijing 100864, China

**Keywords:** RAIM, robust statistics, MM estimator, characteristic slope

## Abstract

The residual-based (RB) receiver autonomous integrity monitoring (RAIM) detector is a widely used receiver integrity enhancement technology that has the ability to rapidly respond to outliers. However, the sensitivity and vulnerability of the residuals to the outliers are the weaknesses of the method especially in the case of multi-outlier modes. It is an effective method for enhancing the validity of residuals by robust estimation instead of least squares (LS) estimation. In this paper, a modified RB RAIM detector based on a robust MM estimation with a higher detection performance under multi-outlier modes is presented. A fast subset selection method based on the characteristic slope that could reduce the number of subsets to be calculated is also presented. The experimental results show that the proposed algorithm maintains a more robust performance than the RB RAIM detector based on the LS estimator and M estimator with an IGG III function especially with the increase in the number of outliers. The proposed fast subset selection method can reduce the calculation time by at least 80%, demonstrating the practical application value of the algorithm.

## 1. Introduction

Integrity is a required feature for global navigation satellite system (GNSS) users. The snapshot receiver autonomous integrity monitoring (RAIM) algorithms based on consistency checks with redundant observations have been widely used especially in aviation [1]. The residual-based (RB) snapshot RAIM schemes are typical post-processing outlier detection methods. Outliers participate in the process of parameter estimation and take the sum of the squares of pseudo-range residuals as the test statistic for outlier detection [2,3]. When a significant deviation exists between the estimated and the actual parameter, the test statistics of the RB RAIM detector can respond quickly and the calculation amount is required. RB RAIM schemes have been developed for aerial navigation where the presence of multiple simultaneous outliers is unlikely. Due to the modernization of American GPS, the full deployment of Russian GLONASS, the emergence of Chinese BDS and European Galileo and the development of regional navigation satellite systems NAVIC and QZSS, more satellites have become available, causing the possibility of double simultaneous satellite failures to be higher than the integrity risk requirement [4,5,6].

For multi-constellation, the probability of three or more simultaneous faults remains an order of magnitude below the integrity requirement [7]. However, the GNSS signal can also be reflected by the multipath effect and non-line-of-sight (NLOS) receptions especially in urban city areas [8,9]. The three or more outliers mode could not be neglected any more. In this case, traditional RB RAIM schemes are invalid as the residuals cannot be used as the basis for outlier identification.

There are two kinds of modified methods for the detection and identification of multiple simultaneous outliers. One is a redesign of the RAIM detector and the other is the optimal parameter estimator. In this paper, we propose solutions aiming at an RB RAIM detector based on a robust estimator. The main contributions of this paper are as follows.

(1) We propose an RB RAIM method based on the MM estimator, which contains a least trimmed squares (LTS) estimator with a high breakdown point and an M estimator with high efficiency, making residuals more consistent with the actual ranging errors. The advantages of the RB RAIM detector are preserved and the ability to detect multiple simultaneous outliers is significantly improved. 

(2) We present a fast subset selection method based on the characteristic slope. With an increasing amount of available observations, the number of subsets that an LTS estimator needs to estimate increases dramatically. The efficiency of robust estimation is improved as much as possible by removing the satellites that make little contribution to the geometry while ensuring a better position dilution of precision (PDOP) condition. 

The remainder of this paper is organized as follows. The related works on RAIM for a multi-outlier mode are given in Section 2. The camouflage effect of an RB RAIM in a multiple outlier mode is analyzed in Section 3. The proposed RB RAIM based on MM estimation and the fast subset selection method are detailed in Section 4. Section 5 describes the experiments used to demonstrate the practical utility of our approach. Finally, the discussion and conclusions are provided in Section 6.

## 2. Related Works

Over the past decade, scholars have done some related work on a modified RAIM algorithm for a multi-outlier mode. The multiple hypothesis solution separation (MHSS) algorithm detects outliers via comparing the position estimate made with all satellites in view with estimates from subsets that have removed some hypothetical outliers [10,11,12,13,14]. The range consensus (RANCO) algorithm calculates position solutions based on the best four-satellite subset and compares this estimate with the pseudo-ranges of all the satellites not contributing to this solution [15,16]. The core idea of this kind of method is to find the best subset that does not contain outliers by relying on a combinatorial search, which could be intractable as the number of visible satellites and hypothetical outliers increases [17,18,19]. Juan et al. [17] modified the MHSS algorithm using a fixed set of subsets to reduce the computational load. Ge et al. [18] presented a modified method that evaluated multiple outlier cases using subsets that excluded entire constellations to narrow the search range for outliers. Other approaches neglect many of the less likely scenarios to simplify the RAIM detector based on the MHSS algorithm [19,20]. These methods only consider outliers caused by satellite failure; in that scenario, the probability of three or more simultaneous outliers is lower than the integrity risk requirement, which can be ignored. The computational burden of these methods increases dramatically as the number of simultaneous outliers increases.

Mathieu et al. [21] designed a detector based on a non-least-squares (NLS) estimator to reduce the integrity risk at the cost of lower accuracy. The concept aimed to improve the combined availability of accuracy and integrity. Hwang and Brown [22] proposed NIORAIM to better balance accuracy and integrity. The weights were designed both in the position domain and in the measurement domain. Song et al. [23] proposed the correlation-weighted least squares residual (CW-LSR) algorithm in which the pseudo-range residuals were weighted with the characteristic slope, making it approximate to the optimal test statistic. However, the characteristic slope was valid only in a single outlier mode. The robust estimators could also improve the robustness and effectiveness of residuals although the accuracy was not optimal. Yang [24] presented an RB RAIM detector based on a robust M estimation with an IGG III function for multiple outlier detection and exclusion, which could also control the influence of near-failure observations. However, the robustness of the M estimation built on the relatively reliable iterative initial value, which was generally the LS estimation, thus having a breakdown point tending to zero especially when the number of simultaneous outliers increased [25,26,27]. Knight and Wang [28] compared the outlier test and robust methods and identified MM estimators [29,30] and the L1 norm obtained the highest rates of normal exclusion as the number of outliers increased. Thus, it is the objective of this paper to improve the ability of an RB RAIM to detect and identify simultaneous outliers based on MM estimation.

## 3. Camouflage Effect of an RB RAIM Detector in Multi-Outlier Mode

### 3.1. Baseline of an RB RAIM Detector

This section introduces notations describing the well-established least squares (LS) estimator and residual-based (RB) RAIM detector and analyzes the camouflage effect of pseudo-range residuals, which is the main factor in reducing the fault detection and exclusion (FDE) performance of an RB RAIM detector in a multiple outlier mode.

Let m and n denote the number of states to be estimated and visible satellites, respectively. Assume the linearize functional model is [8]:

(1) y=Hx+ε+b

where y is an n×1 vector of observations containing the differences between the expected ranging values and the raw ones to all visible satellites, H is an n×(m+3) linear observation matrix, x is an (m+3)×1 state vector that obtains m clock offsets and 3-dimensional (3D) positions in the local Cartesian coordinate (ENU), b is an n×1 bias vector that is a zero vector when there is no outlier among the observations and ε is an n×1 observation error vector, which is assumed to be normally distributed with the zero-mean and covariance matrix D [8]:

(2) ε∼N(0,D)

where 0 is an n×1 zero vector and D is assumed to be diagonal. The probability distribution of nominal observation noise can be reliably modeled using large amounts of experimental data and bounded by the Gaussian function described in Equation (2).

The LS solution for the state vector x in Equation (1) is:

(3) $$\hat{x}={\left(H^{T}WH\right)}^{-1}H^{T}Wy,W=D^{-1}.$$

The correction of the state parameter vector is:

(4) $$\delta \hat{x}=\hat{x}-x={\left(H^{T}WH\right)}^{-1}H^{T}W\left(\varepsilon +b\right).$$

Let $A={\left(H^{T}WH\right)}^{-1}H^{T}W$. We obtain $\delta \hat{x}=A\left(\varepsilon +b\right)$. The pseudo-range residual vector r is:

(5) $$r=y-H\hat{x}.$$

Here, we take a single GNSS as an example and the number of state parameters to be estimated should be added accordingly for multiple GNSSs.

By substituting Equation (4) into Equation (5), the residual vector can be expressed as:

(6) $$r=\left(\varepsilon +b\right)-H\delta \hat{x}=\left(\varepsilon +b\right)-H{\left(H^{T}WH\right)}^{-1}H^{T}W\left(\varepsilon +b\right)=\left(I-H{\left(H^{T}WH\right)}^{-1}H^{T}W\right)\left(\varepsilon +b\right).$$

Let $S=I-H{\left(H^{T}WH\right)}^{-1}H^{T}W$ and r=S⋅(ε+b). The residual vector r can be regarded as the projection of the error vector (ε+b) through the symmetric idempotent matrix S. The residual vector is used as a substitute for FDE because the error vector (ε+b) is unknown.

The Chi-square test statistic of an RB RAIM detector is computed as:

(7) $$T_{RB}=\sqrt{r^{T}Wr/(n-m-3)}∼\chi^{2}(n-m-3,\lambda_{RB}^{2}).$$

The test statistic $T_{RB}$ follows a non-central Chi-square distribution with (n−m−3) degrees of freedom and a non-centrality parameter $\lambda_{RB}^{2}$, which is function of the fault vector b:

(8) $$\lambda_{RB}^{2}=b^{T}DSb.$$

The threshold $T_{D}$ of the Chi-square test can then be calculated by the number of satellites and the possibility of a false alarm. The detector rejects the observation with the largest test statistic in the outlier exclusion. If multiple outliers exist, then the single outlier test is applied iteratively until all outliers have been removed.

Equation (5) shows that if we can obtain an absolutely accurate estimation of the state parameters then we obtain r=(ε+b). However, it is impossible to achieve the expected result. However, the more accurate the estimated state parameters, the more reliable the FDE result of an RB RAIM.

### 3.2. Camouflage Effect of Residuals

The residual vector is the projection of the error vector under matrix S. Equation (6) can be expressed as the following equations:

(9) $$\left[\begin{array}{c} r_{1}\\ r_{2}\\ \vdots \\ r_{n} \end{array}\right]=\left[\begin{array}{cccc} S_{11} & S_{12} & \cdots & S_{1n}\\ S_{21} & S_{22} & \cdots & S_{2n}\\ \vdots & \vdots & \vdots & \vdots \\ S_{n1} & S_{n2} & \cdots & S_{nn} \end{array}\right]\cdot \left(\left[\begin{array}{c} \varepsilon_{1}\\ \varepsilon_{2}\\ \vdots \\ \varepsilon_{n} \end{array}\right]+\left[\begin{array}{c} b_{1}\\ b_{2}\\ \vdots \\ b_{n} \end{array}\right]\right).$$

As the residuals depend on the state parameters estimation, the residual vector r and the linear observation matrix H are also polluted when there are some outliers in observations. An outlier affects the residuals of all observations and the impacts of different outliers are different. The residual of each observation could consist of two parts: one caused by itself, $r_{i,self}$ and the other one caused by other observations, $r_{i,others}$. In the case of no outliers, the bias vector b is a zero vector and the residual of each pseudo-range could be expressed as:

(10) $$r_{i}=S_{i,:}\cdot \varepsilon =S\cdot \varepsilon +\sum_{j=1,j\neq i}^{n}S_{ij}\cdot \varepsilon_{j}=r_{i,self}+r_{i,others}$$

where $r_{i,self}=S_{ii}\cdot \varepsilon_{i}$ and $r_{i,others}=\sum_{j=1,j\neq i}^{n}S_{ij}\cdot \varepsilon_{j}$. In general, $r_{i,self}>r_{i,others}$ as $S_{ii}>S_{ij},j\neq i$ in matrix S. In this case, the presence of $r_{i,others}$ does not have an obvious effect on the FDE performance of an RB RAIM detector.

There are differences when different pseudo-ranges are outliers, which is also known as the leverage effect. Therefore, the studentized residuals are used to replace the residuals. Leverage observations are pseudo-ranges that have a high potential to influence the estimated parameters. The corresponding residual may be unobvious, which may cause masking and swamping under poor geometric conditions.

In the case of a single outlier, let the *g*th observation be the outlier and the bias vector can be expressed as $b={\left[\begin{array}{ccccccc} 0 & \cdots & 0 & b_{g} & 0 & \cdots & 0 \end{array}\right]}^{T}$. The residual of each observation can also consist of two parts: one caused by noise, $r_{i,noise}$ and the other one caused by bias, $r_{i,bias}$.

The residual of the normal pseudo-range $r_{i}$ could be expressed as:

(11) $$r_{i}=\sum_{j=1}^{n}S_{ij}\cdot \varepsilon_{j}+S_{ig}\cdot b_{g}=r_{i,noise}+r_{i,bias}$$

The residual of outlier $r_{g}$ can be expressed as

(12) $$r_{g}=\sum_{j=1}^{n}S_{gj}\cdot \varepsilon_{j}+S_{gg}\cdot b_{g}=r_{g,noise}+r_{g,bias}$$

Matrix S has the following properties:

- It is a symmetric matrix, $S^{T}=S$;
- It is an idempotent matrix, $S^{2}=S$ and $S_{i,:}\cdot S_{:,i}=S_{ii}$; and
- The diagonal elements are $S_{ii}\in \left(0,1\right)$ and $S_{ii}>S_{ij},i\neq j$.

The influence of an outlier on its own residual is greater than that of the residuals of normal observations $\left|r_{g,bias}\right|>\left|r_{i,bias}\right|$; therefore, the outlier may be identified relatively easily by the RB RAIM detector especially when using the studentized residuals. However, in a multiple outlier mode, let the *g*th and *k*th pseudo-range be the two outliers and the residual of the normal pseudo-range $r_{i}$ could be expressed as:

(13) $$r_{i}=\sum_{j=1}^{n}S_{ij}\cdot \varepsilon_{j}+S_{ig}\cdot b_{g}+S_{ik}\cdot b_{k}$$

The residuals of outliers $r_{g}$ and $r_{k}$ can be expressed as:

(14) $$\{\begin{array}{c} r_{g}=\sum_{j=1}^{n}S_{gj}\cdot \varepsilon_{j}+S_{gg}\cdot b_{g}+S_{gk}\cdot b_{k}\\ r_{k}=\sum_{j=1}^{n}S_{kj}\cdot \varepsilon_{j}+S_{kg}\cdot b_{g}+S_{kk}\cdot b_{k} \end{array}$$

At this time, it is not possible to identify outliers by the relationship of the elements of matrix S. The following two factors should be considered.

(1) The combination of outliers. The diagonal elements of S are differentiated depending on the geometry.
(2) The magnitude and direction of outliers. The ratio of two biases is $\beta =b_{g}/b_{k}$, taking two outliers as an example.

Therefore, due to the overlapping of $r_{i,bias}$, the residual of outliers may not be obvious. We define this situation as the camouflage effect of residuals in a multiple outlier mode, which creates difficulties for the performance of outlier identification in the RB RAIM detector.

The camouflage effect causes two types of mistakes in the FDE of the RB RAIM detector: masking and swamping. Masking means the misidentification of outliers so the final state estimation will still be contaminated by outliers. Swamping means the identification of normal observations as outliers so that the accuracy of the final state estimation will be compromised.

## 4. An RB RAIM Based on a Robust MM Estimation

### 4.1. Robust Principle and an RB RAIM Detector Based on a Robust Estimation

The least squares method has been criticized for its dramatic lack of robustness and outliers having an arbitrarily large effect on the residuals by LS estimation. In this connection, Hampel introduced the notion of the breakdown point $\varepsilon^{*}$, which is the smallest percentage of contaminated data that can cause the estimator to take on arbitrarily large aberrant values. In the case of least squares, $\varepsilon^{*}=0$.

To reduce the effect of outliers on parameters estimations, robust estimation methods have been widely used; M estimation is the most popular as it is based on the generalized maximum likelihood estimation theory and is closely connected to traditional LS procedures.

Mitigation of multiple outliers for GNSS positioning has been reported using M estimation. The authors confirmed superior performance of the scheme over a classical LS solution for the scenarios with multiple outliers. The M estimator was first proposed by Huber where the LS cost function of squared residuals was replaced by a symmetric convex cost function ρ:

(15) $$\mathrm{minimize}\sum_{i=1}^{n}w_{i}\rho \left(\frac{r_{i}}{\hat{\sigma }}\right)$$

where $r_{i}$ is the residual of each observation and $\hat{\sigma }$ is the scale factor of the residuals (an estimator of the spread of the random errors). A robust alternative is subtracting the median instead of the mean and then taking the median of the absolute values, which yields the median of absolute deviation (MAD) and the normalized MAD (MADN) as:

(16) $$\hat{\sigma }=MADN(r)=\frac{median\left(\left|r-median(r)\right|\right)}{0.6745}$$

The normalized residual of the *i*th pseudo-range could be formed as $v_{i}=\frac{r_{i}}{\hat{\sigma }}$.

**Figure 1:**
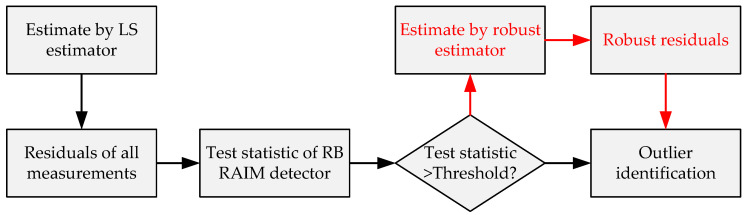
Flow chart of a residual-based (RB) receiver autonomous integrity monitoring (RAIM) detector based on a robust estimator.

The LS estimation makes full use of all pseudo-ranges that include the possible existence of outliers; therefore, the LS residuals are directly affected by outliers. For a robust estimation, the weight of outliers may be reduced in the process of iteration to weaken the influence of outliers on the residuals. The flow chart of the RB RAIM detector based on a robust estimator is shown in Figure 1.

The robust M estimator can be derived as:

(17) $${\hat{x}}_{R}={\left(H^{T}\bar{P}H\right)}^{-1}H^{T}\bar{P}y$$

where $\bar{P}$ is a diagonal equivalent weight matrix and the *i*th diagonal element ${\bar{p}}_{ii}$ of $\bar{P}$ is computed as:

(18) $${\bar{p}}_{ii}=p_{i}w_{i}$$

where $w_{i}$ is the *i*th diagonal element of W. The weight matrix W is replaced by $\bar{P}$ in a robust M estimation.

### 4.2. Robust MM Estimation

When using an MM estimator, the main idea is to have a high breakdown point method that is aggressive toward the elimination of observations, often eliminating more observations that necessary, making it less efficient for obtaining an initial estimate for scale and state. The scale factor is then held constant during consecutive iterations of the M estimator starting with the final robust state parameters.

In this paper, the employed high breakdown point estimation is based on the LTS estimator and the considered refining M estimation is based on the Huber score function. On this basis, a fast subset selection method was designed to optimize the calculation amount of the LTS estimator to improve the practicability of the MM estimator for more available observations.

**Figure 2:**
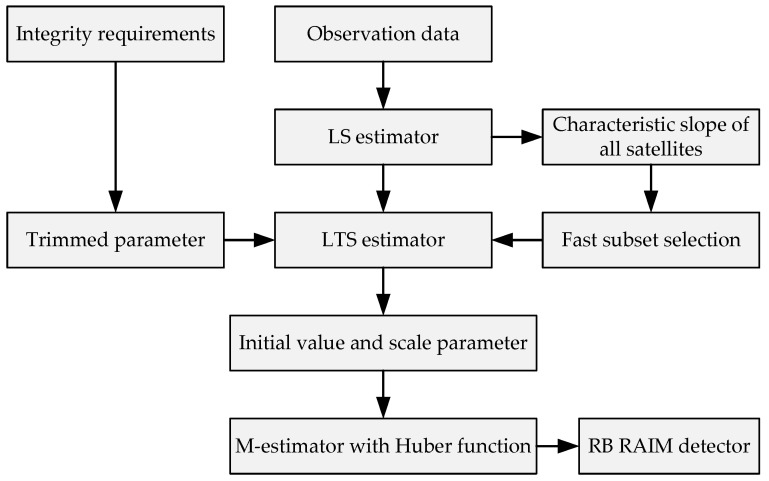
Flow chart of an RB detector based on an MM estimator.

The flow chart of the RB detector based on the proposed MM estimator is shown in Figure 2.

#### 4.2.1. Least Trimmed Squares Estimator

The LTS estimator was developed by Rousseeuw, which is close to ordinary least squares except that the largest weighted squared residuals are excluded from the summation to be minimized [31]. The robust LTS estimation is based on the subset of h cases (out of n) whose least squares fit possesses the smallest sum of squared residuals:

(19) $$\mathrm{minimize}\left\{\sum_{i=1}^{h}r_{\left(i\right)}^{2}\right\}$$

where $r_{\left(i\right)}^{2}$ is the squared residuals in ascending order. Only one subset with the h smallest squared residuals would be considered so that (n−h) pseudo-ranges would not be used in the robust estimation. In this way, the LTS estimation has sufficient robustness when the number of outliers does not exceed (n−h).

The LTS estimation is somewhat similar to the MHSS in principle. Given the trimmed parameter h, the observation model in Equation (1) could be partitioned to distinguish the subset obtaining h observations (subscript A) from the subset, obtaining (n−h) observations (subscript B):

(20) $$\left[\begin{array}{c} y_{A}\\ y_{B} \end{array}\right]=\left[\begin{array}{c} H_{A}\\ H_{B} \end{array}\right]x+\left[\begin{array}{c} \varepsilon_{A}\\ \varepsilon_{B} \end{array}\right]+\left[\begin{array}{c} b_{A}\\ b_{B} \end{array}\right].$$

**Figure 3:**
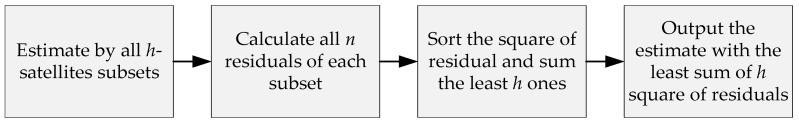
Flow chart of the least trimmed squares (LTS) estimator.

We obtain $C_{n}^{h}=\frac{n!}{h!(n-h)!}$ possible subsets and obtain the corresponding state parameter vector estimation based on LS estimation by the subset with subscript A. The corresponding residuals of all n pseudo-ranges are then computed and the h smallest squared residuals are arranged in ascending order for each subset. The flow chart of the LTS estimator is shown in Figure 3.

The scale factor estimated by the LTS estimator is

(21) $${\hat{\sigma }}_{LTS}={\left(\sum_{i=1}^{h}{\left|v\right|}_{i}^{2}\right)}^{1/2}.$$

From Equation (21), we determine that the breakdown point of the LTS estimator is determined by h. In general, the range of h is n/2≤h<n and the breakdown point is h/n, which should be set to 0.5 to achieve maximum robustness. 

We can adjust the balance between robustness and efficiency by setting an appropriate value for h. Considering the requirements for the number of visible satellites in GNSS positioning:

- At least (m+3) satellites are required to complete the positioning solution. Defining the basic lower limit of the trimmed parameter is the maximum of (n−m−3) and n/2;
- In general, the PDOP decreases as the number of visible satellites increases. The accuracy deteriorates, which affects the effectiveness of the corresponding residuals;
- The value of the trimmed parameter should be as large as possible to avoid too many subsets to be calculated; and
- All possible multiple outlier modes need to be within the effective robust range to ensure the robustness of the estimation, which defines the upper limit of the trimmed parameter according to the integrity requirements.

#### 4.2.2. Equivalent Weight Function of the M Estimation

The M estimation is based on the minimization of a score function of residuals that aim at a result not influenced by outliers. There are more than 10 kinds of score function and the following four representative functions (Cauchy, Huber, Tukey and IGG III) were selected for comparison, as shown in Table 1.

**Figure 4:**
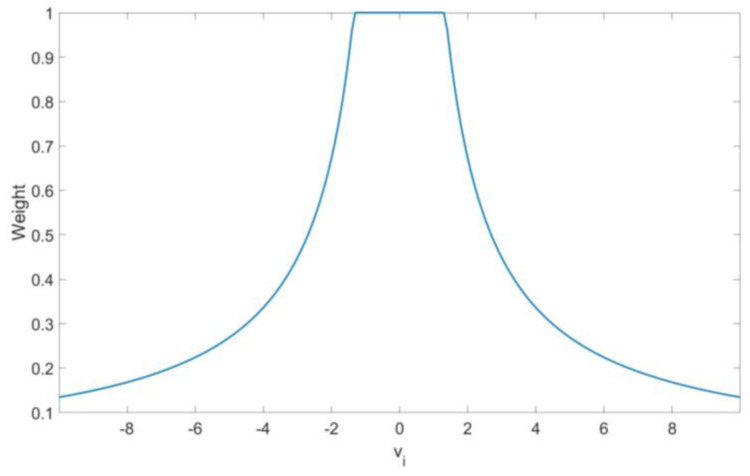
Huber weight function.

A monotone-based M estimator is then applied, which is not as aggressive as the previous one, to neglect some observations but not decrease the efficiency. Therefore, the M estimate after the initial LTS almost always uses the Huber score function. Otherwise, when using a hard-redescending function, such as the Tukey or the IGG III, concatenate an aggressive initial estimate with an also-aggressive posterior one. The Huber function was found to be the best choice of the above four and was adopted in this study, as shown in Figure 4.

### 4.3. Fast Subsets Selection Based on the Characteristic Slope

The LTS estimation requires more operations for sorting the squared residuals than MHSS, which means that LTS estimation creates a larger computational burden for the receiver especially for multiple GNSS users.

When the number of visible satellites reaches a certain value, the improvement of the PDOP by adding additional satellites gradually decreases but the growth of the number of subsets to be calculated gradually increases. A fast subset selection method was designed to determine the satellite with the least contribution to the PDOP in the current set based on the characteristic slope. These satellites are eliminated until the termination condition is reached.

The observation matrix is denoted by $H_{n}={\left[\begin{array}{ccc} h_{1}^{T} & \cdots & h_{n}^{T} \end{array}\right]}^{T}$ and $h_{i},i=1,\ldots ,n$ is the row vector corresponding to each satellite. After removing the *k*th one of all satellites, the observation matrix is $H_{n-1}^{k}={\left[\begin{array}{cccccc} h_{1}^{T} & \cdots & h_{k-1}^{T} & h_{k+1}^{T} & \cdots & h_{n}^{T} \end{array}\right]}^{T}$ and the relationship between $H_{n}$ and $H_{n-1}^{k}$ is:

(22) $$H_{n}^{T}H_{n}=H_{n-1}^{kT}H_{n-1}^{k}+h_{k}^{T}h_{k}.$$

Let $G_{n}={\left(H_{n}^{T}H_{n}\right)}^{-1}$; the PDOP could be calculated as:

(23) $$PDOP=\sqrt{\sum_{i=1}^{3}G_{n}(i,i)}$$

and let $G_{n-1}={\left(H_{n-1}^{kT}H_{n-1}^{k}\right)}^{-1}$. The Sherman–Morrison formula provides a numerical relationship between $G_{n-1}^{k}$ and $G_{n}$:

(24) $$G_{n-1}^{k}={\left(H_{n}^{T}H_{n}-h_{k}^{T}h_{k}\right)}^{-1}=G_{n}+\frac{G_{n}h_{k}^{T}h_{k}G_{n}}{1-h_{k}G_{n}h_{k}^{T}}.$$

Let $K_{k}=(1-h_{k}G_{n}h_{k}^{T})$ and $P_{k}=G_{n}h_{k}^{T}h_{k}G_{n}/K_{k}$. The relationship between $PDOP_{n}$ and $PDOP_{n-1}$ is:

(25) $$PDOP_{n-1}^{k2}=PDOP_{n}^{2}+\sum_{i=1}^{3}P_{k}(i,i).$$

The contribution of the *k*th satellite to the PDOP is then:

(26) $$\sqrt{PDOP_{n-1}^{k2}-PDOP_{n}^{2}}=\sqrt{\sum_{i=1}^{3}P_{k}(i,i)}=\sqrt{\frac{\sum_{i=1}^{m+3}{\left(A_{n}(k,i)\right)}^{2}}{S_{n}(k,k)}}=Slope_{k}.$$

Following Kaplan, the characteristic slope [32] is defined as:

(27) $$Slope_{k}=\sqrt{\frac{\sum_{i=1}^{3}{\left(A_{n}(k,i)\right)}^{2}}{S_{n}(k,k)}}$$

The characteristic slope is an indicator used to measure the degree of leverage in RB RAIM. Equation (27) shows that it also represents the degree of contribution of each satellite to the PDOP. The matrices A and S here are calculated by the observation matrix H. Although there is an error between the matrix H used here and the real value, the position error of the receiver is negligible here because the distance between the receiver and the satellite is usually tens of thousands of kilometers. In this way, we remove the satellite with the lowest characteristic slope from the set that contributes the least to the PDOP but can effectively reduce the number of subsets to be estimated in the LTS estimator.

**Figure 5:**
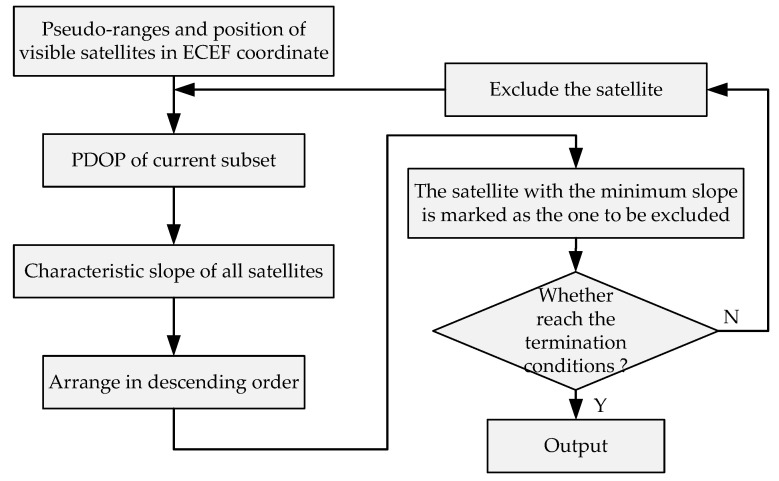
Flow chart of subset selection method based on the characteristic slope.

The main advantage is that we do not need to calculate the PDOP of all (n−1) satellites combinations to identify the satellite that has the least impact on the PDOP, which avoids a large number of repeated complex matrix operations. Only the elements in matrix A and S then need to be used for numerical calculation, which considerably reduces the computation. The flow chart of the subset selection method based on the characteristic slope is shown in Figure 5. Set the termination threshold of the selection iteration, including the threshold λ of the change rate of the PDOP, the threshold ${\mathrm{PDOP}}_{T}$ of the position dilution of precision, the protection threshold $n_{sin}$ of the number of remaining satellites of a single constellation and the protection threshold $n_{mul}$ of the number of remaining satellites of all constellations.

The termination criteria for subset selection include the following aspects:

- If $PDOP_{S}>PDOP_{T}$, the process is terminated and the result is output;
- If ΔPDOP>λ, move the satellite back to S and take the set S as the selected subset, terminate the process and output the result;
- If the number of remaining satellites of the constellation meets the protection threshold $n_{sin}$, mark the constellation as a protected one; if $num_{con\_pro}=num_{con}$, take the current set as the selected subset, terminate the process and output the result;
- If $num_{sat}\leq n_{mul}$, terminate the process and take the current set as the selected subset.

After the proposed fast subset selection, the number of subsets to be calculated by the LTS estimator is reduced by:

(28) $$\Delta num_{subset}=\frac{C_{n}^{h}-C_{n_{sel}}^{h}}{C_{n}^{h}}=\frac{n!\left(n_{sel}-h\right)!-n_{sel}!\left(n-h\right)!}{n!\left(n_{sel}-h\right)!}.$$

With the decrease in h, the effect of the proposed fast subset selection on the reduction of computation is more obvious. However, the number of satellites in each subset decreases accordingly so the termination threshold of the subset selection can be appropriately relaxed.

## 5. Algorithm Verification and Simulation Results

### 5.1. Simulation Conditions

**Figure 6:**
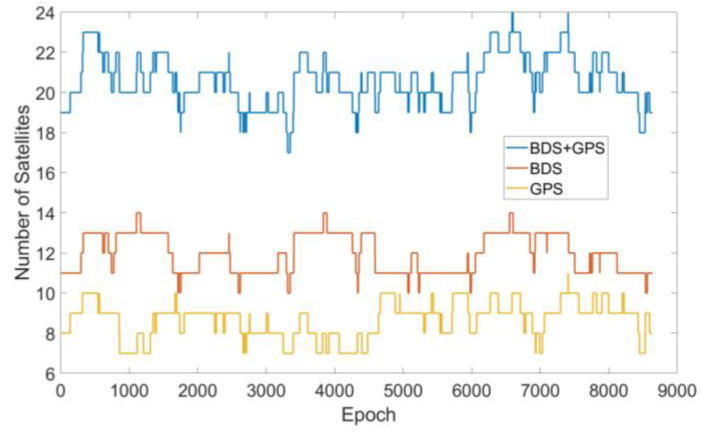
The number of satellites during simulation.

To compare the RB RAIM detectors, 24 h of GPS and BDS data were simulated. The orbit data of the 210th day of 2020 were download from CELESTRACK with a 10° masking angle and the observation update rate was 10 Hz. The simulated receiver was set on the roof of the Aerospace Information Research Institute, Chinese Academy of Sciences (Lat. 40°4′12″, Lon. 116°16′12″, height 100 m). As simulation hardware an Intel Core i5-10210U CPU @2.60 GHz with 16 GB RAM was used and all simulations were performed within MATLAB 2019a. The number of visible satellites during simulation is displayed in Figure 6.

The pseudo-range noises follow the same uncorrelated Gaussian distribution and the *i*th element of diagonal covariance matrices $\sigma_{i,i}^{2}$ satisfies:

(29) $$\sigma_{i,i}^{2}=\sigma_{URA,i}^{2}+\sigma_{tropo,i}^{2}+\sigma_{user,i}^{2}$$

where $\sigma_{URA,i}^{2}$ is the user range accuracy (URA) of the *i*th satellite, which was set to 0.75 m [2]. The tropospheric delay model and the user error model are shown in Table 2.

Four RB RAIM detectors were compared:

- Detector 1: Baseline RB RAIM detector based on the LS estimator.
- Detector 2: RB RAIM detector based on the M estimator with IGG III function with the constant values $k_{0}=1.5$ and $k_{1}=3.0$.
- Detector 3: RB RAIM detector based on the MM estimator; the trimmed parameter was h=n−4.
- Detector 4: RB RAIM detector proposed in this paper; the trimmed parameter was h=n−4 and the termination criteria were $n_{\sin}=6$, $n_{mul}=12$ and λ=0.03.

**Figure 7:**
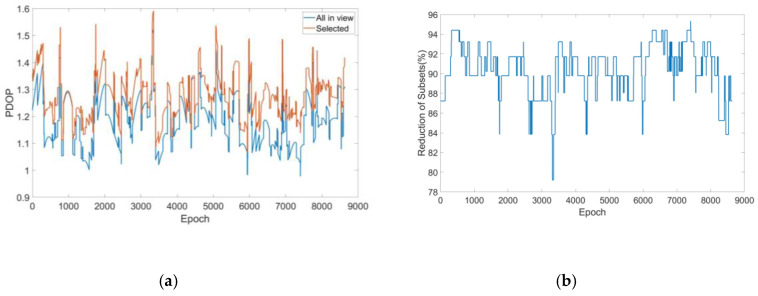
(**a**) Comparison of the position dilution of precision (PDOP); (**b**) reduction of subsets after subset selection.

The PDOP value and the reduction of subsets to be calculated during simulation by the fast subset selection method based on the characteristic slope are shown in Figure 7. The mean value of the PDOP for the all-in-view and selected were 1.1928 and 1.3617, respectively, which increased by 14.16% after subset selection but this was still within a relatively good range. However, at this time, the number of corresponding subsets to be calculated decreased over 90%, which considerably reduced the calculation amount for the LTS estimator.

The simulation consisted of the following three cases:

Case 1: Each combination of two outliers was considered with a fixed bias in the first epoch of simulation.

Case 2: A comparison of the detectors for the double outliers combination with the largest characteristic slope in each sampling epoch with a fixed β=1. 

Assume that the *i*th and *j*th satellite are two failures and $b_{j}=\beta b_{i}$ according to the definition of the characteristic slope:

(30) $$Slope_{ij}=\sqrt{{\frac{{\left(A_{1i}+\beta A_{1j}\right)}^{2}+{\left(A_{2i}+\beta A_{2j}\right)}^{2}+\left(A_{3i}+\beta A_{3j}\right)}{S_{ii}+\left(1+\beta \right)S_{ij}+\beta S_{jj}}}^{2}}.$$

Case 3: A comparison of the detectors for random one, two, three and four outliers with a random bias in each epoch separately. 

### 5.2. Comparison of Double Outlier Combinations with a Fixed Bias

**Figure 8:**
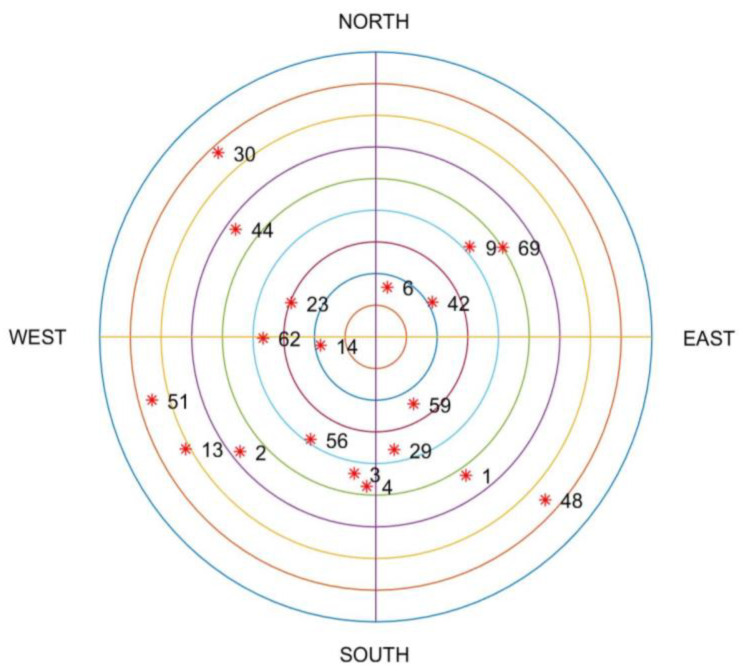
The status of satellites at the beginning of simulation.

The status of the visible satellites at the beginning of the simulation is shown in Figure 8. BDS satellites are numbered from PRN 1 to 30 and GPS satellites are numbered from PRN 41 to 71.

**Figure 9:**
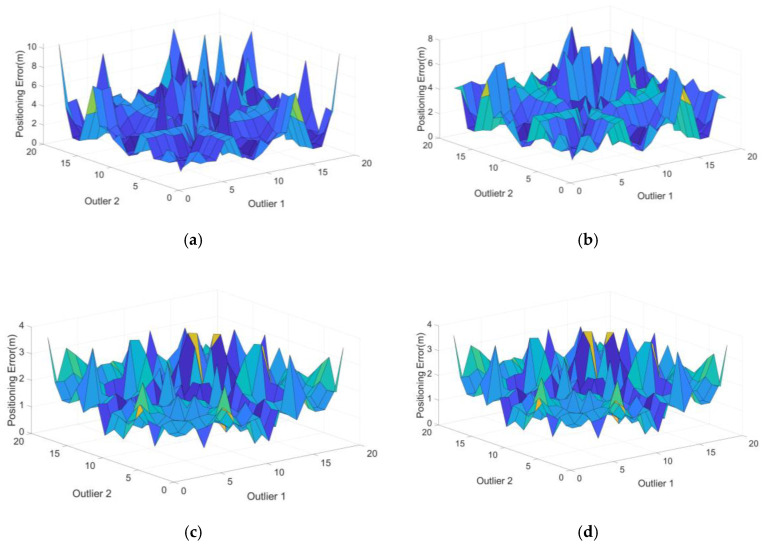
Positioning errors of four detectors for all combinations in Case 1: (**a**) detector 1, (**b**) detector 2, (**c**) detector 3 and (**d**) detector 4.

At the initial epoch of simulation, there were 19 visible satellites and 171 double outlier combinations were obtained. Bias was fixed to 10 m and the positioning errors of the four RB RAIM detectors after outlier elimination are shown in Figure 9.

Compared with detector 1, detector 2 produced a better detection effect for individual but not all combinations of double outliers as shown in Figure 9a,b. This showed that the M estimator could be robust but without a high breakdown point. Figure 9c,d showed that there was no obvious difference between the positioning errors of detector 3 and detector 4. The numerical results extracted from the detectors (Table 3) included the RMSE of the positioning error, maximum and 95% confidence level errors and the simulation time.

The simulation time of detector 4 reduced by 80.71% compared with detector 3 and there was no significant difference in the accuracy of the final parameter estimation with obvious advantages compared with detectors 1 and 2.

### 5.3. Double Outlier Mode with the Largest Characteristic Slope

**Figure 10:**
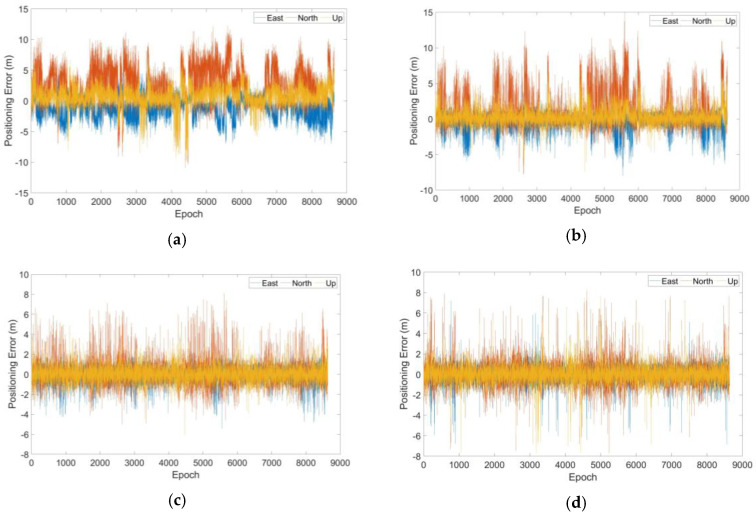
Positioning errors of four detectors for the worst combination in Case 2: (**a**) detector 1, (**b**) detector 2, (**c**) detector 3 and (**d**) detector 4.

For Case 2, a fixed bias of 10 m was added into the double outliers combination with the largest characteristic slope with β=1 at each epoch during simulation. The positioning errors (ENU) of the four detectors are shown in Figure 10.

**Figure 11:**
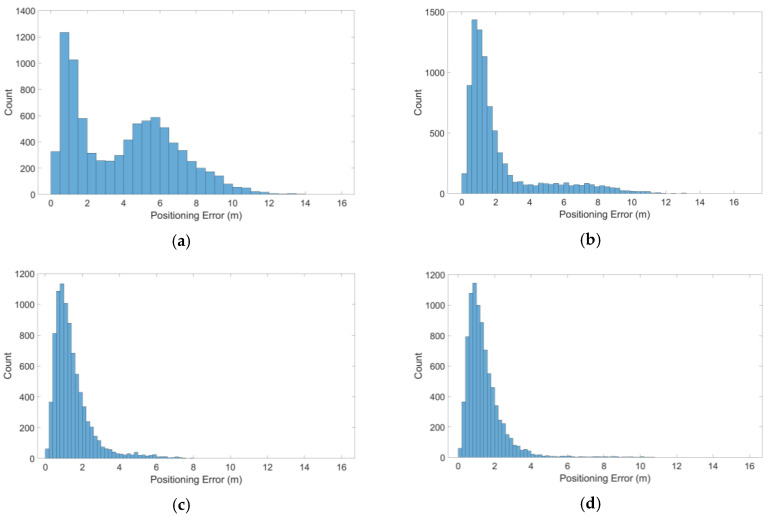
3D positioning errors of the four detectors for the worst combination in Case 2: (**a**) detector 1, (**b**) detector 2, (**c**) detector 3 and (**d**) detector 4.

In Figure 10, the M estimator in detector 2 did not produce an effective robust effect compared with the MM estimator in detectors 3 and 4. The accuracy of detector 4 was slightly poorer than that of detector 3 but the positioning result obtained was robust. The distribution statistics of 3D positioning errors of the four detectors are shown in Figure 11. The numerical results extracted from the detectors in Case 2 are shown in Table 4.

The simulation time of detector 4 was reduced by 82.33% compared with detector 3 by using the proposed fast subset selection method. Compared with the simulation time in Table 2, the proposed fast subset selection based on the characteristic slope was effective. Considering the considerably improved efficiency of estimation, sacrificing some robustness within a reasonable limit was acceptable.

### 5.4. Detection and Exclusion for a Multiple Outlier Mode

Outliers were inserted into the pseudo-ranges and the success rates of the outlier test and 3D positioning errors after outlier exclusion were recorded. Based on the comparison of the pseudo-ranges excluded and the pseudo-ranges that contained simulated outliers, the estimated parameters were categorized as shown in Table 1. The scenarios were:

(a) None of the outliers were excluded and some normal pseudo-ranges were excluded;
(b) Some of the outliers were excluded;
(c) Some of the outliers were excluded and some normal pseudo-ranges were also excluded;
(d) All of the outliers were excluded and some normal pseudo-ranges were also excluded; and
(e) All of the outliers were excluded and none of the normal pseudo-ranges were excluded.

**Figure 12:**
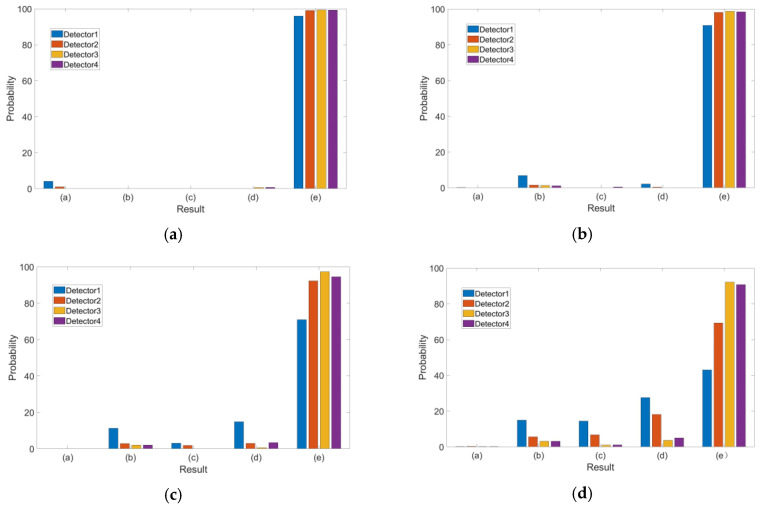
Identification result of four detectors in Case 3: (**a**) one, (**b**) two, (**c**) three and (**d**) four outliers.

For Case 3, a bias of 10 m was randomly added into one to four outlier combinations at each epoch during simulation. The detection results of the four algorithms are shown in Figure 12 and the statistical results are provided in Table 5.

**Figure 13:**
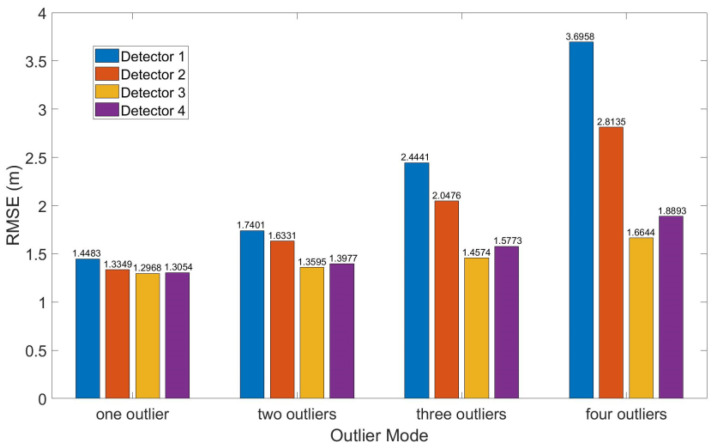
Root mean squared error (RMSE) of four detectors in Case 3.

The root mean squared error (RMSE) of the four algorithms are shown in Figure 13. The results of detector 4 were similar to those of detector 3 especially when the number of outliers was less than four.

As the number of outliers increased, the amounts of categories (b) and (c) increased while categories (d) and (e) of the correct exclusion decreased for all detectors and especially for detector 1. For detector 2, the probability of category (d) decreased rapidly with the increase in outliers so that many normal observations were excluded although outliers were excluded. However, for detectors 3 and 4, the probability of categories (d) and (e) were still over 90% although the probability of category (e) also decreased with the increase in outliers.

Category (d) could be also an acceptable result when there were enough available pseudo-ranges compared with the misidentification of any outlier.

## 6. Discussion and Conclusions

In this paper we first analyzed the residuals that could not be used as an effective basis for outlier identification when there were multiple simultaneous outliers, which is called the camouflage effect of residuals. In this case, replacing the LS estimator by the M estimator could not effectively improve the identification rate of multiple outliers as the robustness of the M estimation is built on the basis of a relatively reliable iterative initial value. We proposed an RB RAIM algorithm based on the MM estimator, which contained an LTS estimator with a high breakdown point and an M estimator with a Huber function with high efficiency. We designed a fast subset selection method based on the characteristic slope to improve the practicability of the algorithm as the computation amount of the LTS estimator increased sharply with the increase in the number of visible satellites. The simulated experimental results demonstrated that the proposed RB RAIM detector based on the MM estimator maintained a more robust performance than the LS estimator and the M estimator with an IGG III function especially with the increase of the number of outliers. The fast subset selection method based on the characteristic slope reduced the calculation time by more than 80%, which indicated the practical application value of the algorithm.

Notably, categories (d) and (e) could be regarded as a correct exclusion; however, a high amount of category (d) was generally undesirable as it could lead to the positioning solution being unavailable if the number of satellites was small. The exclusion of normal observations was less than desirable because all normal observations would not be used. Although the probabilities of categories (d) and (e) were still more than 90%, with the increase of outliers, the actual detection effect of detector 3 and 4 gradually deteriorated. However, this became less of a concern as the number of visible satellites increased.

Future work can focus on further reducing the computational burden. The subset selection process is not necessary in each epoch so we can design a judgment threshold to determine the validity period of each selected subset. In addition, the inertial information of the receiver can be used to provide support to determine the reliable initial iteration value for a robust estimation [33]. However, this may cause a new potential integrity risk, which requires further research and experimentation. 

## Figures and Tables

**Table 1:** Four representative score functions.

Scheme	Function	Parameter
Cauchy	${\bar{p}}_{i}=\frac{1}{1+{\left(\frac{\left|v_{i}\right|}{k}\right)}^{2}}$	k=2.3849
Huber	${\bar{p}}_{i}=\{\begin{array}{cc} 1, & \left|v_{i}\right|<k\\ \frac{k\sigma }{\left|v_{i}\right|}, & \left|v_{i}\right|\geq k \end{array}$	k=1.3450
Tukey	${\bar{p}}_{i}=\{\begin{array}{cc} {\left(1-{\left(\frac{v_{i}}{k}\right)}^{2}\right)}^{2}, & \left|v_{i}\right|<k\\ 0, & \left|v_{i}\right|\geq k \end{array}$	k=4.6851
IGG III	${\bar{p}}_{i}=\{\begin{array}{cc} 1, & \left|v_{i}\right|<k_{0}\\ \frac{k_{0}}{\left|v_{i}\right|}\cdot {\left(\frac{k_{1}-\left|v_{i}\right|}{k_{1}-k_{0}}\right)}^{2}, & k_{0}\leq \left|v_{i}\right|<k_{1}\\ 0, & \left|v_{i}\right|\geq k_{1} \end{array}$	$\begin{array}{l} k_{0}=1.0∼2.0\\ k_{1}=2.5∼3.5 \end{array}$

**Table 2:** Error model.

Error	Function	Parameter
Tropospheric delay	$\sigma_{tropo,i}=0.12\cdot 1.001/\sqrt{0.002001+\sin{\left(\theta_{i}\right)}^{2}}$	$\theta_{i}$ is the elevation angle
User error	$\sigma_{user,i}=\sqrt{\sigma_{MP}^{2}+\sigma_{noise}^{2}}\sqrt{f_{1}^{4}+f_{2}^{4}}/(f_{1}^{2}-f_{2}^{2})$	For GPS: $f_{L1}=1575.42\;\mathrm{MHz}$, $f_{L2}=1227.60\;\mathrm{MHz}$ For BDS: $f_{B1}=1561.098\;\mathrm{MHz}$, $f_{B2}=1207.14\;\mathrm{MHz}$
Multiple path	$\sigma_{MP}=0.13+0.53\exp(-18\theta_{i}/\pi )$	
Noise	$\sigma_{noise}=0.15+0.43\exp(-180\theta_{i}/6.9/\pi )$	

**Table 3:** The numerical results of the four detectors in Case 1.

	RMSE (m)	95% (m)	Max (m)	Time (s)
Detector 1	3.6112	5.8041	10.5030	10.7667
Detector 2	3.2587	5.1707	7.5235	11.5730
Detector 3	1.8418	2.6215	3.6394	173.8353
Detector 4	1.8418	2.6215	3.6394	33.5331

**Table 4:** The numerical results of four detectors in Case 2.

	RMSE (m)	95% (m)	Max (m)	Time (s)
Detector 1	4.8796	8.8160	13.8602	231.6745
Detector 2	3.1876	7.7496	16.1177	298.7104
Detector 3	1.7912	3.4720	8.5700	4711.9302
Detector 4	1.8132	3.4850	11.2629	832.5141

**Table 5:** The statistical results of four detectors in Case 3.

	**One Outlier**				**Two Outliers**			
	(a)	(b) + (c)	(d) + (e)	(e)	(a)	(b) + (c)	(d) + (e)	(e)
Detector 1	4%	0%	96%	96%	0%	7%	93%	91%
Detector 2	1%	0%	99%	99%	0%	2%	98%	98%
Detector 3	0%	0%	100%	99%	0%	1%	99%	99%
Detector 4	0%	0%	100%	99%	0%	1%	99%	98%
	**Three Outliers**				**Four Outliers**			
	(a)	(b) + (c)	(d) + (e)	(e)	(a)	(b) + (c)	(d) + (e)	(e)
Detector 1	0%	14%	86%	71%	0%	29%	71%	43%
Detector 2	0%	6%	94%	91%	0%	13%	87%	69%
Detector 3	0%	2%	98%	97%	0%	4%	96%	92%
Detector 4	0%	2%	98%	95%	0%	4%	96%	91%
